# Association of Oxidative Balance Score with the Metabolic Syndrome in a Sample of Iranian Adults

**DOI:** 10.1155/2021/5593919

**Published:** 2021-05-26

**Authors:** Zahra Noruzi, Ahmad Jayedi, Mena Farazi, Elaheh Asgari, Fatemeh Dehghani Firouzabadi, Zahra Akbarzadeh, Kurosh Djafarian, Sakineh Shab-Bidar

**Affiliations:** ^1^Department of Community Nutrition, School of Nutritional Sciences and Dietetics, Tehran University of Medical Sciences (TUMS), Tehran, Iran; ^2^Department of Clinical Nutrition, School of Nutritional Sciences and Dietetics, Tehran University of Medical Sciences (TUMS), Tehran, Iran

## Abstract

**Objective:**

We aimed to assess the association of the oxidative balance score (OBS) with metabolic syndrome (MetS) in adults.

**Design:**

A population-based cross-sectional study *Setting*. Health centers from five districts in Tehran, Iran.

**Methods:**

We recruited 847 participants with an age range of 18-65 years. Dietary intake was assessed by a semiquantitative food frequency questionnaire with 168 items. The OBS was calculated by using the following 13 dietary and nondietary anti- and prooxidant components: dietary antioxidants (selenium, fiber, *β*-carotene, vitamin D, vitamin C, vitamin E, and folate), dietary prooxidants (iron and saturated and polyunsaturated fatty acids), and nondietary anti- (physical activity) and prooxidants (smoking and obesity). The odds ratio (OR) and 95% confidence interval (CI) of the MetS and its components across tertiles of the OBS were calculated by logistic regression analysis, controlling for age, sex, energy intake, occupation, and educational level.

**Results:**

The range of OBS was between 16 and 39. Being in the top versus the bottom tertile of the OBS was not associated with the MetS (OR = 0.71, 95% CI 0.48-1.03; *P* = 0.07), after controlling for potential confounders. Higher OBS score was associated with a lower likelihood of abdominal obesity (OR: 0.55, 95% CI: 0.38-0.81; *P* = 0.003) and increased diastolic blood pressure (OR: 0.64, 95% CI: 0.41-0.99; *P* = 0.04). Higher OBS was not associated with other components of the MetS.

**Conclusion:**

Overall, the present study showed that there was no significant relationship between OBS and MetS in Tehranian adults.

## 1. Introduction

The metabolic syndrome (MetS) is a collection of several metabolic disorders including abdominal obesity, dyslipidemia, hypertension, hyperglycemia, inflammation, high body mass index (BMI), and insulin resistance [1]. The incidence of MetS often occurs in parallel with the incidence of obesity and type 2 diabetes [2]. The prevalence of MetS is about 20-30% of the adult's population in developed countries [3]. The occurrence of MetS and obesity is increasing in Iran [4, 5]. Around one-third of adults in Iran are affected by MetS [6].

MetS is a consequence of a complex interaction between several factors such as unhealthy dietary patterns and lifestyle-related factors [2, 7]. It has been proposed that oxidative stress may be involved in the pathogenesis and progression of MetS [8]. Obesity seems to exacerbate the body's oxidant/antioxidant balance in favour of free radicals and oxidative stress [9–11]. As body weight increases, the ability of the antioxidant defence system decreases to stop the activities of free radicals, so multifactorial metabolic disorders such as hypertension will appear [12]. Free radicals can damage DNA, proteins, and lipids, leading to cell injury and unfavourable health outcomes like tissue injury that is related to vascular damage [13]. There is evidence that higher dietary antioxidant intake can mitigate levels of oxidative stress and metabolic disorders, namely, diabetes and inflammation [14, 15]. Researchers have presented that a combination of antioxidant components can be more strongly associated with metabolic disorders than any individual antioxidant nutrient [16]. In addition to anti- and prooxidant dietary components, other lifestyle-related behaviours such as cigarette smoking [17], physical activity [18], and adiposity [19] can affect levels of oxidative stress in the body. So to capture the whole oxidant/antioxidant properties of lifestyle, Goodman et al. introduced an oxidative balance score (OBS) as a measure of combined pro- and antioxidant exposure status of an individual, with a higher score indicating a predominance of anti- over prooxidant exposures [20, 21]. Several studies have investigated the association between OBS and various chronic diseases like different cancer types and cardiovascular diseases and inflammation [13, 22–24].

Considering the role of oxidative stress in the development of MetS [25], this study was aimed at examining the association of the OBS, a proxy of combined anti- and prooxidant properties of the diet and lifestyle, with the risk of MetS in a sample of Tehranian adults, hypothesizing that a higher OBS would be associated with a lower risk of MetS.

## 2. Methods

### 2.1. Study Participants

The sample size required for this study was calculated by considering the frequency of obesity as a dependent variable using the following formula. Taking into account the total prevalence of overweight and obesity of 68.5% in Tehran adults, with 95% confidence and a maximum estimation error of 5% and a value of 0.04 as the effect size, the sample size was 546.

(1) $$\begin{array}{c} n=\frac{z^{2}\cdot p\left(1-p\right)}{d^{2}}=\frac{{\left(1/96\right)}^{2}\times 0/65\times 0/35}{{\left(0/04\right)}^{2}}=546. \end{array}$$

Due to the fact that cluster sampling is two-stage and the response of individuals in each health center may be correlated, the number of samples is multiplied by the effect factor of the cluster design selected as 1.5, and the total number of samples 819 is obtained, which we consider as 850, because this data is taken from the previous design and the effect of dietary pattern on obesity is more sensitive than its effect on cardiometabolic and inflammatory biomarkers; in the present study, the sample size of 850 is considered.

Eight hundred fifty adult males and females referred to the health care centers in different zones of Tehran took part in the present cross-sectional study. Five zones including North, South, West, East, and the center of the city were selected. Next, a list of all health care centers that existed in each zone was provided. We randomly chose eight health centers from five districts (40 health centers) and then divided the total sample size (850) by the number of health centers (40) to obtain the number of participants in each health center. Inclusion criteria were apparently healthy male and female, aged between 20 and 59 years, living in Tehran city, who attended the local health care centers during the study period (2018-2019), and had a willingness to take part in the study.

### 2.2. Ethical Approval

All participants gave written informed consent before their inclusion in the study. The study was performed in accordance with the Declaration of Helsinki, and the procedure of the study was approved by the Research Ethics Committee of Tehran University of Medical Science (Ethic Number: IR.TUMS.VCR.REC.1397.157). Detailed written information regarding the background and procedures of the study was presented to participants.

### 2.3. Demographic Factors

At the first visit at each health center, data about gender (male, female), age, education (under diploma, diploma), smoking status (current smoking, former smoking, never smoking), occupation (employed, retried, housekeeper, or unemployed), and marital status (married or other) were collected by trained interviews through prespecified data extraction forms.

### 2.4. Physical Activity

The validated International Physical Activity Questionnaire (IPAQ) was utilized for determining physical activity levels [26]. Metabolic equivalent (MET) was used for calculating the level of physical activity [27]. Participants were categorized into two groups including very low (<600 MET-minute/week) or low (600-3000 MET-minute/week) and moderate and high physical activity (>3000 MET-minute/week).

### 2.5. Anthropometric and Blood Pressure Assessments and Laboratory Tests

A wall stadiometer with a sensitivity of 0.1 cm (Seca, Germany) was used for measuring the participants' height. They were asked to take off their shoes for height measurement. Participants' weight was measured, without coat and jacket, by using an adult's digital scale (808Seca, Germany) with 0.1 kg precision. BMI was calculated as weight in kilograms divided by height in meters squared. Waist circumference (WC) was measured with a tape measure to the nearest 0.1 cm between the iliac crest and the lowest rib during exhalation. After 10 to 15 minutes of rest and sitting, systolic (SBP) and diastolic (DBP) blood pressure was measured by a trained physician, on the right arm, with a digital barometer (BC 08, Beurer, Germany). For each participant, the mean of two blood pressure measurements was recorded. Fasting plasma glucose (FPG), serum triglyceride (TG), and high-density lipoprotein cholesterol (HDL-C) were measured after 10–12 hours of overnight fasting from a venous blood sample. Blood samples were measured by standard methods at the Nutrition and Biochemistry Laboratory of the School of Nutritional Sciences and Dietetics at Tehran University of Medical Sciences.

### 2.6. Dietary Assessment

A semiquantitative food frequency questionnaire with 168 items [28] was used for assessing the average dietary intake during the past year. The frequency eating (daily, weekly, monthly, and annual) of each food in the food frequency questionnaire was recorded in an interview by trained dieticians. Collected data were converted into g/d according to household measures by using Nutritionist IV software based on the Iranian food-modified US Department of Agriculture food composition database [29].

### 2.7. Definition of Metabolic Syndrome

The diagnostic criteria introduced by NCEP ATP III [30] was used to define cardiometabolic abnormalities, and accordingly, the metabolic syndrome was established when three or more of the following disorders were presented: (1) central obesity as WC ≥ 102 cm in men and ≥88 cm in women; (2) hypertriglyceridemia as serum TG ≥ 150 mg/dL (1.69 mmol/L); (3) HDL − C < 40 mg/dL (1.04 mmol/L) for men and <50 mg/dL (1.29 mmol/L) for women; (4) hypertension as systolic/diastolic blood pressure ≥ 130/85 mmHg or taking antihypertensive medications; and (5) hyperglycemia as FPG ≥ 100 mg/dL (5.6 mmol/L) or taking antidiabetic medications.

### 2.8. Oxidative Balance Score

We used the method introduced by Goodman et al., to calculate the OBS of each participant. According to this method, a total of 13 dietary and nondietary pro- and antioxidant components, based on a priori knowledge about their association to oxidative stress, were selected [21]. The components were divided into four groups: (1) dietary antioxidants (selenium, fiber, *β*-carotene, vitamin D, vitamin C, vitamin E, and folate); (2) dietary prooxidants (iron and saturated (SFA) and polyunsaturated (PUFA) fatty acids); (3) nondietary antioxidant (physical activity); and (4) nondietary prooxidants (smoking and obesity). Dietary factors were ranked into tertiles. For dietary antioxidants and physical activity, the first to third tertiles were assigned scores of 1-3. An inverse scoring was used for dietary prooxidants. For obesity, we assigned 1: BMI ≥ 30 kg/m^2^ and WC ≥ 102 cm in males and ≥88 cm in females, 2: either BMI ≥ 30 kg/m^2^ or WC ≥ 102 cm in males or ≥88 cm in females, and 3: BMI < 30 kg/m^2^ and WC < 102 in males or <88 cm in females. For smoking, we assigned 1: current smoking, 2: former smoking, and 3: never smoking. The score of four components was then summed to calculate the OBS for each participant. The minimum and maximum scores possible are, respectively, 3 and 39.

### 2.9. Statistical Analysis

The OBS was categorized into tertiles, and then, the characteristics of the participants were presented accordingly. Continuous variables were presented as mean and standard deviation (SD) and categorical variables as frequency (number) across tertiles of the OBS and then compared by using the one-way analysis of variance (ANOVA) and the chi-square tests, respectively. The odds ratio (OR) and 95% confidence interval (CI) of MetS and its components were estimated through binary logistic regression analysis in two models. Model I adjusted for age, sex, and energy intakes as potential confounders. Model II was adjusted for confounders in Model I plus education and occupation status. All statistical analyses were done by the use of SPSS software version 24 (IBM, Armonk, NY). *P* value less than 0.05 was considered statistically significant.

## 3. Results

The initial sample size included 850 participants; of those, three participants were excluded due to lack of data for at least one variable. Therefore, 847 participants were included in the present study. Characteristics of the participants and dietary intake across tertiles of the OBS are indicated in Table 1. The mean age of participants was 44.7 ± 10.7 years, of whom 31.3% were men. The prevalence of MetS was 30.5%. The range of the OBS was between 16 and 36. Compared to those in the lowest tertile of the OBS, participants in the highest tertile were more likely to have lower BMI and WC and lower iron intake. Besides, those with a higher level of OBS had a higher intake of vitamins C, D, and E, fiber, beta-carotene, and folate. There were no significant differences in gender, age, smoking status, and dietary intake of selenium, PUFA, and SFA across tertiles of the OBS.

Table 2 indicates the biochemical variables of the participants across tertiles of the OBS. There were no significant differences in terms of biochemical variables across tertiles of the OBS.  Table 3 represents the OR and its 95% CI of MetS and its components across tertiles of the OBS. The results showed that there was no significant association between OBS and MetS. The OR of MetS for the third versus first tertile of the OBS was 0.71 (95% CI: 0.48, 1.03; *P* = 0.07) in the fully adjusted model. There were significant relationships between higher levels of OBS and abdominal obesity as assessed by WC (OR = 0.55, 95% CI: 0.38, 0.82; *P* = 0.003) and increased DBP (OR: 0.64, 95% CI: 0.41, 0.99; *P* = 0.04). There was no significant association between the OBS and other components of the MetS.

## 4. Discussion

Oxidative stress is associated with several health outcomes like diabetes, inflammation, different types of cancer, and other metabolic disorders [13, 15, 22]. To the best of our knowledge, no previous study has been done to investigate the relationship between this score and MetS in Iran. In this cross-sectional study of Tehranian adults, we assessed the status of oxidative balance, represented by OBS, and its association with MetS. There was no significant association between OBS and MetS either in crude or in the fully adjusted models. There was also no significant relationship between OBS and components of the MetS, except for increased WC and DBP.

Current evidence regarding the association of a priori OBS and the risk of chronic diseases is inconsistent. Two prospective cohort studies showed that a balanced oxidative status was associated with a lower risk of all-cause and cause-specific mortality [24, 31]. Some case-control studies suggested an inverse association between the OBS and the likelihood of colorectal and prostate cancers [20, 21, 32]. One large cohort study found an inverse association between the OBS and colorectal cancer risk [33], whilst two other case-cohort studies suggested no association between the OBS and prostate cancer risk [34, 35].

Evidence regarding the association of the OBS and the MetS is scarce. Our preliminary search found only one study that assessed the association of oxidant/antioxidant disequilibrium and MetS [36]. A recent cross-sectional study among 6400 Korean adults aged >40 years showed that those in the fourth quartile of the OBS had 35% lower odds of the MetS as compared to those in the first tertile. They included dietary anti- (vitamin C, carotene, and retinol) and prooxidants (iron) and nondietary components including smoking status, physical activity, and alcohol drinking to calculate the OBS [36]. The inconsistency between our findings and the Korean cross-sectional study may be due to different sample sizes and adjustment models and different components of the OBS in each study. Due to cultural reasons, we did not have sufficient data regarding alcohol drinking to include in the OBS. Also, different dietary components were used to calculate the OBS in two studies.

Another cross-sectional study in Thailand showed that those with the MetS had lower circulating levels of superoxide dismutase, catalase, and vitamin C than those without the MetS [37]. However, they used serum biomarkers to evaluate oxidant/antioxidant disequilibrium.

We also found no association between the OBS and most components of the MetS. Exceptions were increased DBP and WC, for which inverse associations were found. Our results regarding the null association between the OBS and high fasting glucose and SBP and inverse associations with increased DBP and WC are consistent with those of the Korean cross-sectional study [36]. However, in contrast to their findings, we found no association between OBS and the likelihood of hypertriglyceridemia and low HDL-C concentration.

Another cross-sectional evaluation within a prospective cohort study in the US found inverse associations between the OBS and increased WC and low-density lipoprotein cholesterol concentration, and no association for total cholesterol and TG [38]. The dietary components used to calculate the OBS in that study were the intake of vitamin C, *α*-carotene, *β*-carotene, *β*-cryptoxanthin, lutein, lycopene, selenium, iron, and PUFA, as well as nondietary factors including smoking history, alcohol drinking, and the use of aspirin and other nonsteroid anti-inflammatory drugs.

Although we did not find a significant relation between OBS and the MetS, several biological pathways suggest a link between oxidative stress and cardiometabolic disorders [13]. Recent studies suggest that a high level of oxidative stress may increase the risk of MetS by activating proinflammatory mediators [39] and several transcriptional, molecular, and metabolic pathways. Oxidative stress is an underlying pathologic mechanism of insulin resistance [40, 41] and thereby can exert unfavourable impacts in the pathways of glucose metabolism, blood pressure, and serum lipid abnormalities [42]. Oxidative stress is accompanied by the more production of reactive oxygen species. The reactive oxygen species can induce lipid peroxidation, which in turn is associated with several cardiometabolic abnormalities [43]. In addition to the toxic effects of the reactive oxygen species, they can decrease the endothelial production of nitric oxide [12]. Studies found that levels of nitric oxide in obese patients who have hypertension were lower than those without hypertension [12, 44].

One of the strengths of our study was the ability to include both dietary and lifestyle components into the score, so a comprehensive view of different determinants of oxidative stress was provided. Our study included a relatively large sample size involving both men and women. In addition, data was collected by expert dieticians with valid and reliable questionnaires. On the other hand, our study has some limitations which can affect the results. Because of cross-sectional design and the temporal sequence, as a result, a casual association cannot be concluded. Moreover, some items were missing in the OBS (i.e., alcohol drinking). Also, the known limitations of FFQ for dietary assessment, i.e., the error in recall and limited food choices, should be considered. Finally, we had a lack of information about genotypes that can affect OBS and components of the MetS [38]. We also did not include endogenous factors which may affect oxidative stress [15].

## 5. Conclusions

In conclusion, no significant association between OBS and odds of MetS in adults was found. However, a significant inverse association was found between OBS and abdominal adiposity and DBP. Further studies, in particular those with prospective nature, need to confirm the present findings.

## Figures and Tables

**(a):** 

Characteristics^∗^	T1 [16–24] (*n* = 278)	T2 [25–28] (*n* = 311)	T3 [29–39] (*n* = 258)	*P* value^∗∗^
Gender (%)				0.68
Male	38.3%	33.8%	27.8%	
Female	30.3%	38%	31.7%	
Education (%)				0.18
Illiterate	27.8%	37.5%	34.7%	
Under diploma	35%	33.6%	31.4%	
Diploma	31.5%	33.8%	34.6%	
Educated	33.6%	41.4%	25%	
Smoking (%)				0.007
Current	50%	22.7%	27.3%	
Former	50%	17.6%	32.4%	
Never	31.1%	38.4%	30.6%	
Physical activity (MET-min/day)				<0.001
Low	37.4%	35.4%	27.2%	
Medium or high^a^	24.8%	39%	36.1%	

**(b):** 

	Mean	SD	Mean	SD	Mean	SD	
Age (y)	44.3	10.1	44.5	10.7	45.3	11.4	0.27
BMI (kg/m^2^)	28.4	6.9	27.8	4.9	27.4	4.5	0.04
WC (cm)	93.6	13.1	91.2	11.9	91.4	12.2	0.04
Dietary intake							
Energy (kcal/d)	2408	904	2504	1048	2561	906	0.80
Iron (mg/d)	20.2	9.05	20.6	9.44	22.15	8.25	0.016
SFA (mg/d)	25.1	14.8	24.8	17.9	24.6	18.9	0.75
PUFA (mg/d)	17.0	9.4	17.5	13.5	16.8	4.9	0.90
Selenium (*μ*g/d)	0.02	0.04	0.04	0.28	0.03	0.04	0.46
Fiber (g/d)	12.2	4.8	23.9	1.0	34.5	88.4	0.003
*β*-Carotene (*μ*g/d)	402	336	805	657	1319	838	<0.001
Vitamin D (*μ*g/d)	0.83	1.22	1.80	1.97	3.20	3.45	<0.001
Vitamin E (mg/d)	2.68	1.56	4.44	3.31	5.83	3.00	<0.001
Folate (*μ*g/d)	214	83	333	273	503	335	<0.001
Vitamin C (mg/d)	90	40	144	71	213	76	<0.001

Abbreviations: BMI: body mass index; MET: metabolic equivalent; OBS: oxidative balance score; PUFA: polyunsaturated fatty acid; SFA: saturated fatty acid; T: tertile; WC: waist circumference. ^∗^Data are presented as a percentage for categorical variables and mean ± standard deviation for continuous variables. ^∗∗^The one-way analysis of variance and the chi-square test, respectively, were used for comparison of continuous and categorical variables among tertiles of the OBS. *P* < 0.05 is statistically significant. ^a^Because the number of people with high physical activity was low, we combined them with moderate physical activity.

**Table 2:** Biochemical variables of the study participants across tertiles of the oxidative balance score.

	T1		T2		T3		*P* value^∗^
	Mean	SD	Mean	SD	Mean	SD	
Fasting plasma glucose (mg/dL)	108.0	36.5	111.0	38.6	105.0	57.7	0.51
Total cholesterol (mg/dL)	192.0	48.3	200.0	43.1	195.0	42.1	0.44
HDL-C (mg/dL)	32.3	25.9	35.1	23.8	36.0	24.5	0.08
Triglyceride (mg/dL)	144.0	77.1	149	81.8	141.0	79.2	0.64
Triglyceride/HDL ratio	23.5	66.9	12.8	47.9	15.5	56.8	0.17

Abbreviations: HDL-C: high-density lipoprotein cholesterol; T: tertile. ^∗^The one-way analysis of variance was used for comparison of variables among tertiles of the oxidative balance score. *P* < 0.05 was statistically significant.

**Table 3:** The association between oxidative balance score and metabolic syndrome in Tehranian adults (odds ratio and 95% confidence interval).

Oxidative balance score	T1	T2	T3
Cut points of tertiles	16-24	25-28	29-39
Participants (*n*)	278	311	258
High WC			
Crude	1.0	0.98 (0.71-1.35)	0.77 (0.55-1.08)
*P* value^∗^		0.89	0.13
Model I	1.0	0.82 (0.57-1.18)	0.56 (0.38-0.82)
*P* value		0.286	0.003
Model II	1.0	0.85 (0.59-1.22)	0.55 (0.38-0.81)
*P* value		0.37	0.003
High FPG			
Crude	1.0	1.21 (0.84-1.75)	0.73 (0.48-1.10)
*P* value		0.30	0.13
Model I	1.0	1.21 (0.83-1.74)	0.73 (0.48-1.10)
*P* value		0.32	0.13
Model II	1.0	1.20 (0.83-1.74)	0.72 (0.48-1.10)
*P* value		0.33	0.13
High serum TG			
Crude	1.0	1.07 (0.77-1.49)	0.80 (0.56-1.14)
*P* value		0.680	0.22
Model I	1.0	1.06 (0.76-1.48)	0.80 (0.56-1.14)
*P* value		0.72	0.21
Model II	1.0	1.07 (0.77-1.50)	0.78 (0.56-1.14)
*P* value		0.67	0.21
High serum total cholesterol			
Crude	1.0	1.17 (0.84-1.62)	1.19 (0.84-1.68)
*P* value		0.351	0.32
Model I	1.0	1.15 (0.83-1.60)	1.17 (0.83-1.65)
*P* value		0.41	0.38
Model II	1.0	1.12 (0.80-1.56)	1.17 (0.83-1.66)
*P* value		0.50	0.37
Low serum HDL-C			
Crude	1.0	0.88 (0.62-1.26)	0.87 (0.60-1.25)
*P* value		0.49	0.44
Model I	1.0	1.08 (0.72-1.60)	1.06 (0.70-1.61)
*P* value		0.712	0.778
Model II	1.0	1.07 (0.72-1.60)	1.07 (0.70-1.62)
*P* value		0.73	0.76
High systolic blood pressure			
Crude	1.0	1.22 (0.85-1.74)	1.09 (0.74-1.59)
*P* value		0.28	0.66
Model I	1.0	1.18 (0.79-1.78)	0.90 (0.58-1.38)
*P* value		0.41	0.63
Model II	1.0	1.20 (0.80-1.82)	0.91 (0.59-1.40)
*P* value		0.38	0.66
High diastolic blood pressure			
Crude	1.0	1.04 (0.71-1.51)	0.72 (0.47-1.09)
*P* value		0.85	0.12
Model I	1.0	1.01 (0.68-1.50)	0.64 (0.41-0.98)
*P* value		0.96	0.04
Model II	1.0	1.02 (0.68-1.51)	0.64 (0.41-0.99)
*P* value		0.94	0.04
MetS			
Crude	1.0	0.96 (0.68-1.36)	0.71 (0.49-1.04)
*P* value		0.83	0.08
Model I	1.0	0.95 (0.67-1.35)	0.71 (0.48-1.03)
*P* value		0.79	0.07
Model II	1.0	0.96 (0.67-1.36)	0.71 (0.48-1.03)
*P* value		0.82	0.07

Abbreviations: FPG: fasting plasma glucose; HDL: high-density lipoprotein; LDL: low-density lipoprotein; MetS: metabolic syndrome; TG: triacylglycerol; WC: waist circumference. ^∗^*P* values from Mantel-Haenszel extension test. Model I: adjusted for age, sex, and energy intake; Model II: additionally adjusted for occupation and educational level.

## Data Availability

The data support the findings of this study and are available from the corresponding author upon reasonable request.

## Abbreviations

BMI: Body mass index
MET: Metabolic equivalent
OBS: Oxidative balance score
PUFA: Polyunsaturated fatty acid
SFA: Saturated fatty acid
WC: Waist circumference
HDL-C: High-density lipoprotein cholesterol
FPG: Fasting plasma glucose
LDL: Low-density lipoprotein
MetS: Metabolic syndrome
TG: Triacylglycerol.
